# Quantitative Study of the Nonlinearly Enhanced Photoacoustic/Photothermal Effect by Strong LSPR-Coupled Nanoassemblies

**DOI:** 10.3390/nano10101942

**Published:** 2020-09-29

**Authors:** Yujiao Shi, Dandan Cui, Zhenhui Zhang

**Affiliations:** 1 MOE Key Laboratory of Laser Life Science & Institute of Laser Life Science, South China Normal University, Guangzhou 510631, China; 2020010191@m.scnu.edu.cn (D.C.); 2019010190@m.scnu.edu.cn (Z.Z.); 2 Guangdong Provincial Key Laboratory of Laser Life Science, College of Biophotonics, South China Normal University, Guangzhou 510631, China

**Keywords:** photoacoustic imaging, nanoassemblies, photothermal effect, nonlinear effect

## Abstract

The extensive exploration of the collective optical and thermal effects for localized surface plasmon resonance (LSPR)-coupled nanoassemblies has propelled much recent research and development in fields of photoacoustic (PA) imaging and photothermal (PT) therapy, while the rational design and proper engineering of these assemblies under quantitative guidance is still a highly challenging task. In this work, by utilizing the finite element analysis (FEA) method and taking gold nanochains as example, the authors quantitatively studied the coupling optical/thermal response of the nanoassemblies and the associated nonlinearly enhanced PA/PT effect. Results show that compared with their individuals, the strong electromagnetic/thermal coupling between the individuals of the nanoassemblies results in a several-time enhancement of the per-particle-weighted optical absorption, consequential thermal field enhancement, and initial PA pressure, resulting in nonlinearly amplified energy conversion from incident light to heat and PA waves. The dependence of the nonlinear PA/PT enhancement on the assembly chain length, the size of the individuals, the interparticle distance, and the size uniformity of the building blocks is quantitatively discussed. PA experiments on gold nanochains and gold nanospheres are performed to validate the proposition, and the experiments well silhouetted the theoretical discussion. This work paves the way for the rational construction and optimization of plasmonic nanoassemblies with improved PA/PT conversion efficiency.

## 1. Introduction

Subwavelength metallic nanoparticles have been intensively researched for the past decades in fields of energy conversion [1], optical sensing [2], and biomedicine [3], with a unique host of new properties that are notably distinct from their bulk metals [4,5]. The most striking character of these metallic nanoparticles is their unique optical abilities to support localized surface plasmon resonance (LSPR) that arises from the free electrons oscillations [6]. When the metallic nanoparticles are illuminated by light with a wavelength much larger than the nanoparticle size, the high-density free electrons in the metal immediately respond to the external electromagnetic (EM) field and oscillate coherently at the same frequency as the incident light. This plasmon-medicated EM-nanoparticle interaction results in strong light scattering and local EM field enhancement in the vicinity of the nanoparticle surface, which has enabled a variety of applications such as LSPR-enhanced Raman spectroscopy and biological sensing [7,8,9]. As the optical properties of metallic nanoparticles can be fine-tuned by morphology engineering, the LSPR-induced appearance of intense light absorption bands at the tissue’s optical window also fueled the wide exploration of the metallic nanoparticles (e.g., gold nanospheres, nanorods, and nanoshells) as highly perspective exogenous probes in biomedical and biological applications, especially for photoacoustic (PA) imaging and photothermal (PT) therapy [10,11,12,13]. Despite the considerable promising future of metallic nanoparticles and the wide application of plasmon-medicated nanoparticles in PA/PT fields, significant hurdles such as the limited ability to tune the interactions of EM with individual nanoparticles and to further improve their optical and thermal performance still remain.

Self-assembling of the metallic nanoparticles is a new growing point for constructing high-performed LSPR-enhanced nanoprobes in biological applications [14,15,16]. These nanoassemblies can exhibit a rich variety of novel and useful nonlinear effects arising from collective behaviors that are radically different from their individuals in the aspect of optical and thermal responses, which essentially originate in the strong coupled optical and thermal effects among the individual nanoparticles, when the interparticle distance is less than or comparable with the nanoparticle size [17,18]. Therefore, rationally harvesting the coupling effect of the nanoassemblies can maximize our ability to manipulate the performance of their optical and the consequential thermal properties, hence enabling preferable PA/PT bioapplications. Among the most important factors that determine the future trajectory of the application of these assembling metallic nanoparticles, a fundamental understanding, especially a quantitative analysis of the influence of the degree of self-assembly and the morphology, such as the shape, size, interparticle distance on the PA/PT efficiency, is a critical issue. However, even though experimental use of nanoassemblies such as gold nanovesicles has been demonstrated in PA/PT applications [19,20,21,22,23], related quantitative research on this issue still remains unavailable.

Our motivation is to quantitatively explore the LSPR-medicated nonlinearly enhanced PA/PT effect by self-assembling, thus guiding the rational engineering of significantly efficient PA/PT nanoassemblies. Since the structure of chemically synthesized nanoassemblies is usually mathematically complicated, analytical investigation on modelling the coupled EM, thermal fields, and the corresponding nonlinear PA/PT effects is typically impossible. In this work, by utilizing the finite element analysis (FEA) method and taking the basic structured nanosphere-assembled gold nanochains (GNCs) as example, we quantitatively studied the self-assembling-amplified nonlinear optical and thermal responses of the strong LSPR-coupled nanoassemblies, where the quantitative temperature field distribution and the initial PA pressure were obtained. Results show that under a certain pulsed laser illumination, a strong EM/thermal coupling between the individual nanoparticles results in a several-time enhancement of the per-particle-weighted optical absorption, consequential thermal field enhancement, and initial PA pressure, thus inducing nonlinearly amplified energy conversion from incident light to PA/PT effect. The dependence of the nonlinear PA/PT enhancement on the nanoassemblies’ chain length, the size of the individuals, the interparticle distance, and the size uniformity of the building blocks were quantitatively discussed. PA experiments on GNCs and gold nanospheres were performed to validate our proposition, and the experiments have well silhouetted the theoretical discussion. This work paves the way for the rational construction and optimization of plasmonic nanoassemblies with improved PA/PT conversion efficiency.

## 2. Results

The LSPR-mediated optical absorption of laser pulses and the resulting thermal responses, such as heat accumulation and thermoelastic PA wave production, are of particular interest for the fields of biological and biomedicine [24]. The light–matter interaction between the incident pulsed laser and GNCs involves a coupled physical process that spans multispatiotemporal scales, where a rigorous theoretical analysis requires a multiscale analysis that encompasses both quantum and continuum-level theory. Here, in order to simplify the problem, we readily assumed that the continuum theory can be rationally used for exploring the PA wave production process. Furthermore, the FEA computational method is employed as a numerical discretization and solution technique to provide quantitative simulations of the collective LSPR effect, the temperature increase, and the PA signal production processes.

**Figure 1:**
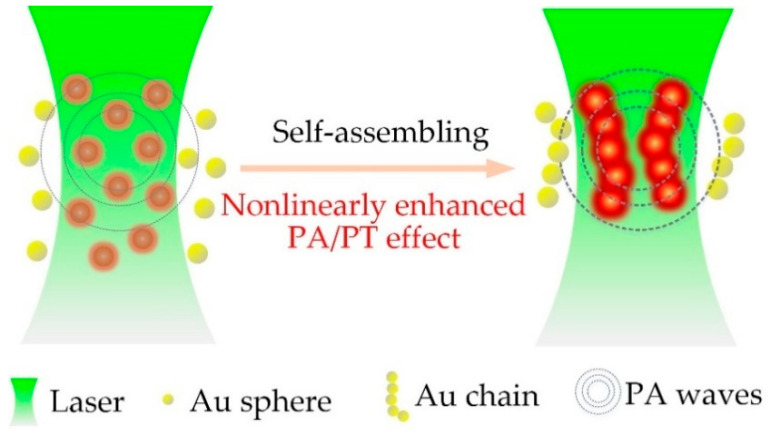
The schematic diagram of the nonlinearly enhanced photoacoustic/photothermal (PA/PT) effect for nanoassemblies.

Figure 1 shows the schematic diagram of the nonlinearly enhanced PA/PT effects for nanoassemblies.

The pronounced optical absorption of the GNCs arises from both the LSPR absorption of both the isolated gold nanosphere and the extremely strong interparticle coupling effects. When the GNCs are irradiated by the incident pulsed laser, free electrons in GNCs quickly jump to high energy levels above the Fermi level by photon–electron interactions within a sub-picosecond [25]. Then, the excited electrons strongly couple between individuals and eventually release their kinetic energy through nonradiative transition by electron–phonon interactions [26]. Assuming that the GNCs are irradiated by a time-harmonic electric field **E** with the frequency of *ω* and intensity of *E_0_*, the electric field distribution in and around the GNCs can be described using the Helmholtz equation [27,28]:

(1) $$\nabla \times \left(\mu_{r}^{-1}\nabla \times E\right)-k_{0}^{2}\left(\varepsilon_{r}-j\frac{\sigma }{\omega \varepsilon_{0}}\right)E=0$$

where $\mu_{r}$, $\varepsilon_{r}$, σ, and $k_{0}$ are the relative permeability, permittivity, conductivity, and the wavenumber, respectively. The strong interparticle coupling between the isolated nanoparticles in the GNCs can be theoretically described by the coupled dipole equation [29,30]:

(2) $$P_{j}=\alpha_{j}{Ε}_{j}=\alpha_{j}\left({Ε}_{inc,j}-\underset{k\neq j}{\sum }A_{jk}P_{jk}\right)$$

where **P*_j_*** represents dipole moments of point dipoles located at positions *r_j_*; *α_j_* is the dipole polarizabilities; and **E*_j_*** is the local electric field, which is the difference of the incident field and the retarded induced field by all other electric dipoles. **A*_jk_*** is a 3 × 3 matrix for j≠k, which can be written as the following [30]:

(3) $$A_{jk}=\frac{exp\left(ikr_{jk}\right)}{r_{jk}}\left[k^{2}\left(\hat{r_{jk}}\hat{r_{jk}}-M_{3}\right)+\frac{ikr_{jk}-1}{r_{jk}{}^{2}}\left(3\hat{r_{jk}}\hat{r_{jk}}-M_{3}\right)\right]$$

with k=ω/c, and $r_{jk}=\left|r_{j}-r_{k}\right|$, $\hat{r_{jk}}=\left(r_{j}-r_{k}\right)/r_{jk}$.$\;M_{3}$ is the 3 × 3 identity matrix. By solving the coupled dipole equations, the absorption cross section of the interparticle coupled GNCs with the total dipole number of *N* can be evaluated as:

(4) $$C_{abs}=\frac{4\pi k}{{\left|E_{0}\right|}^{2}}\overset{N}{\underset{j=1}{\sum }}\left\{Im\left[P_{j}\times {\left(\alpha_{j}{}^{-1}\right)}^{*}P_{j}{}^{*}\right]-\frac{2}{3}k^{3}{\left|P_{j}\right|}^{2}\right\}$$

The interaction between the electric dipoles is quite complicated, and it is hard to obtain an analytical solution for it. In this study, a FEA-based computational method is employed as a quantitative technique to investigate the LSPR-mediated electric field coupling and the enhanced optical absorption. For gold nanoparticles, the permittivity is frequency-dependent and complex-valued, which can be calculated from an analytical model developed by Rioux et al. [31].

**Figure 2:**
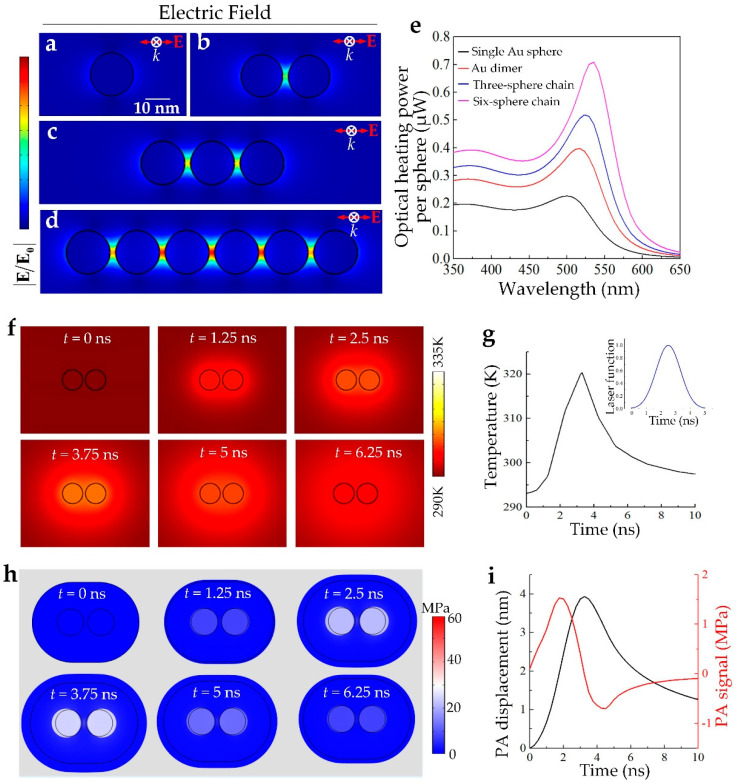
(**a**–**d**) Localized surface plasmon resonance (LSPR)-induced electric field enhancement for gold nanochains (GNCs) with different chain lengths; (**e**) Calculated absorbed power as a function of laser wavelengths for GNCs with different chain lengths; (**f**) Thermal fields of the GNCs with time. The zero time point is the starting time point of the laser pulse; (**g**) The temperature of the gold nano-dimer as a function of time; (**h**) Thermal expansion induced stress of the GNCs and surroundings; (**i**) The resulting PA displacement and signal from the GNCs and surroundings.

Here, in order to provide a systematic understanding of the resonance coupling between each isolated nanoparticle and their contribution to optical absorption, four basic coupling nanostructure cases, i.e., an isolated nanosphere, a nanosphere dimer, a three-nanosphere chain, and a six-nanosphere chain, are studied. The diameter of the gold nanosphere is set to be 15 nm, and the interparticle distance is set to be 2 nm. The incident laser is polarized parallel to the chain axis indicated by red arrows. As shown in Figure 2a–d, compared with the isolated nanosphere, the interaction of the incident electromagnetic radiation with the coupling nanostructures induces strong polarization of the free electrons and dipolar oscillations of the free electrons. For the cases in Figure 2b,c, when the interparticle distance is much less than the nanoparticle diameter, the near-field interaction arising from evanescent fields is the main coupling mechanism [32]. For the case in Figure 2d, where more nanospheres on a longer GNC are coupled, both the near-field and far-field interactions (arising from the propagating dipolar field) are involved and contribute to the electric field enhancement as well as the optical absorption.

Therefore, the six-nanosphere chain displays the highest LSPR coupling effect and electric field enhancement. The collective resonance of the electric dipoles then converts the EM energy into heat through electron–phonon interactions, where the produced thermal power can be quantitatively calculated in terms of resistive heating [33]:

(5) $$Q_{abs}=J\times E,$$

with **J** as the current density resulting from the free electron oscillations. Here, a plane electric wave with the amplitude of $E_{0}=4.6\times 10^{4}$ V/m is induced to irradiate the GNCs and to quantitatively calculate their optical absorption by the FEA method. The calculated incident laser power is about 0.15 mW/μm^2^ according to the irradiance expression $I_{in}=c\varepsilon_{0}n_{sur}E_{0}^{2}/2$, where c, $\varepsilon_{0}$, and $n_{sur}$ are the light speed, vacuum permittivity, and refractive index of the surrounding environment, respectively. In our simulations, we set the GNCs to be immersed in water. Figure 2e shows the calculated optical heating power per sphere as a function of the incident laser wavelength for the isolated nanosphere, the nanosphere dimer, the three-nanosphere chain, and the six-nanosphere chain, respectively, where the highest absorption wavelengths are defined as the LSPR wavelengths. Once the absorbed power per sphere is obtained, the laser energy absorbed by the different coupling nanostructures can be calculated by multiplying the laser pulse width. From Figure 2e we can find that, owing to the interparticle resonance coupling, the peak absorption per sphere gradually grows with the chain length, where the per-particle-weighted optical absorption of the six-nanosphere chain is about 3.5 times higher than that of the isolated nanosphere. Meanwhile, as the chain length grows, the LSPR wavelength shows an obvious red shift from about 505 to 545 nm. The results indicate that, by utilizing the interparticle LSPR coupling through constructing GNCs, the per-particle-weighted optical absorption can be greatly enhanced.

After the GNCs convert the absorbed laser energy into heat, this induces a temperature increase of the GNCs and their surrounding environment owing to the heat diffusion from GNCs to the surroundings, where the temperature field can be described by the heat diffusion equation [34]:

(6) $$\rho C_{p}\frac{\partial T}{\partial t}=k\nabla^{2}T+Q_{abs}\times f\left(t\right),$$

where ρ is the density, *C_p_* is the heat capacity, T is temperature, k is thermal conductivity, $Q_{abs}$ is the produced thermal power by optical energy, and *f*(*t*) is the temporal function of the incident laser pulse. In this study, we assumed that the laser temporal function can be regarded as a Gauss function with a pulse width of τ=5 ns and a standard deviation of σ=τ/6, as $f\left(t\right)=exp\left(-{\left(t-\tau /2\right)}^{2}/2\sigma^{2}\right)/\sqrt{2\pi }\sigma$. We quantitatively simulated the temperature distribution for the nanosphere dimer at different time points after it was irradiated by a pulsed laser with a laser irradiance of 0.15 mW/μm^2^ to simulate the nonlinear PA/PT effects for GNCs. As shown in Figure 2f, when the laser pulse starts to irradiate the GNCs, their temperature gradually increases and then quickly decreases after the laser pulse ends, owing to the thermal diffusion from the GNCs to their surrounding environment. The temperature of the GNCs as a function of time is presented in Figure 2g, where the inset is the laser temporal function.

The local heating of the GNCs by the pulsed laser then induces thermal expansion of the heated area, followed by the production of PA waves, where the produced PA signals can be described by the thermoelastic displacement [35]:

(7) $$\nabla p=-\rho \partial^{2}u/\partial t^{2}$$

which can be usually transformed into $p=-\rho c_{L}\partial u/\partial t$. Here, p, $c_{L}$, and u are the PA pressure, the sound velocity, and the thermoelastic displacement, respectively. Figure 2h shows the simulated thermal expansion and induced stress of the GNCs and their heated surroundings, where obvious thermal-induced volume expansion is observed. By quantitatively extracting the thermal expansion induced PA displacement as a function of time, we obtained the quantitative PA pressure according to Equation (7), shown in Figure 2i. Simulation indicates that under the laser irradiation with an irradiance of 0.15 mW/μm^2^ and a laser pulse of 5 ns, the produced in situ PA pressure peak can reach up to ~MPa.

**Figure 3:**
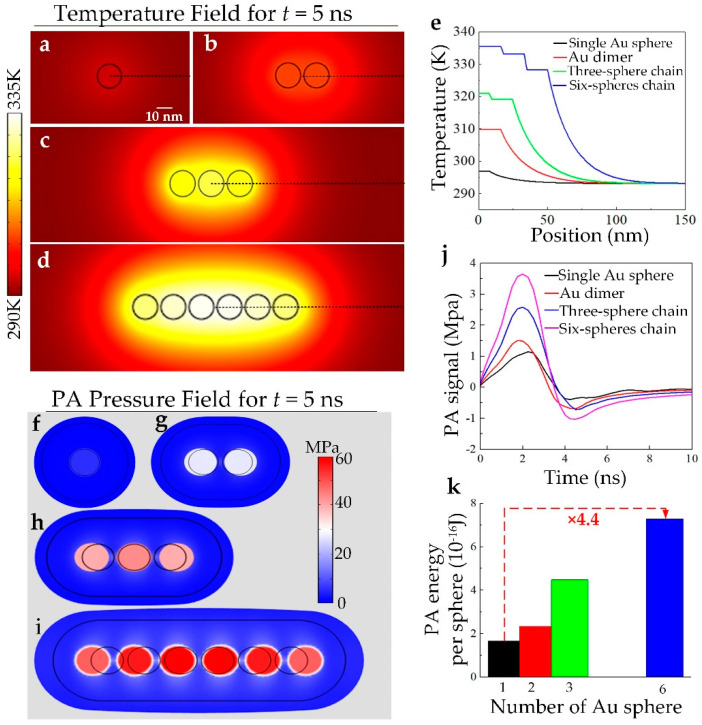
Quantitative thermal fields and PA effect of the GNCs for different chain lengths when irradiated by a laser pulse with an irradiance of 0.15 mW/μm^2^. (**a**–**d**) The thermal fields of the GNCs for different chain lengths at *t* = 5 ns; (**e**) Temperature distribution along the dotted lines in (a–d); (**f**–**i**) The thermal expansion induced stress of the GNCs and surroundings at *t* = 5 ns; (**j**) The corresponding produced PA signals for GNCs with different chain lengths; (**k**) Quantitative calculation of the per-sphere-weighted PA energy for GNCs with different chain lengths.

To demonstrate the nonlinearly enhanced PT effect of the GNCs, we quantitatively simulated the temperature field at the end of the laser pulse for the isolated nanosphere, nanosphere dimer, three-nanosphere chain, and six-nanosphere chain, respectively, in order to simulate the GNCs with different chain lengths, as shown in Figure 3a–d. Under the same laser irradiation, the six-nanosphere chain GNCs exhibit much higher temperature increase compared with other coupling nanostructures, where two main aspects contribute to this phenomenon. One aspect originates in the relatively large per-particle-weighted optical absorption for the six-nanosphere chain compared with other coupling nanostructures. Meanwhile, owing to the heat diffusion from GNCs to the surroundings arising from the small-size effect, as the chain length grows, the interparticle temperature field overlap contributes significantly to the nonlinear local heating of the GNCs. Figure 3e provides spatial temperature distribution for the coupling nanostructures in Figure 3a–d along the black dotted lines. It can be found from Figure 3e that under a certain laser pulse irradiation, the strong LSPR-coupled GNCs exhibits much higher temperature increase. Furthermore, the corresponding thermal induced volume expansion and stress of the GNCs for the four kind of GNCs are quantitatively simulated as shown in Figure 3f–i. Results show that an obvious thermal expansion is observed for all kinds of GNCs, where the six-nanosphere GNCs exhibit much larger PA displacement and volume expansion compared with other lengths of GNCs. By quantitatively extracting the PA displacement as a function of time, the authors obtained the quantitative PA pressure for the four kinds of GNCs according to Equation (7). As shown in Figure 3j, as the length of the GNCs grows larger, the simulated PA pressure gradually increases, where the PA pressure for the six-nanosphere GNCs is about 3 times higher than that of the individuals.

In order to quantify the nonlinear PA effect for the four kinds of GNCs, we calculated the produced PA wave power for one laser pulse, where the total PA energy of the GNCs can be calculated according to the calculation formula of ultrasound power [36]:

(8) $$W_{total}=\frac{p_{eff}{}^{2}}{\rho c_{L}}\times S\times \tau$$

Here, $p_{eff}$ is the effective value of the PA pressure, and S is the surface area of the thermal expansion volume, where we reasonably defined *S* as the surface area in FEA simulation. The per-particle-weighted PA energy $\bar{W}$ for one laser pulse is shown in Figure 3k, where the per-particle-weighted PA energy for the six-nanosphere GNCs is amplified about 4.4 times compared with that of the individuals, even under the same laser irradiance. The results indicate that the self-assembling of metallic nanoparticles can nonlinearly enhance the PA/PT effect, owing to the synergy of both optical and thermal coupling effects between the individuals in the GCNs, where with the length of the GNCs growing larger, the nonlinear enhancement increases.

**Figure 4:**
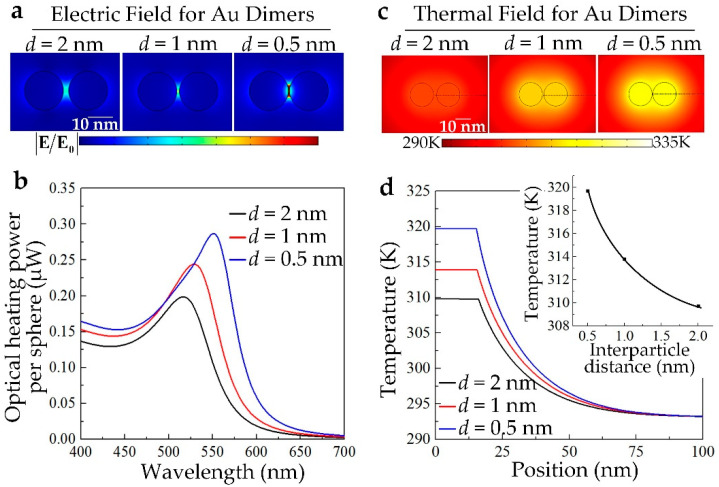
(**a**) LSPR-induced electric field enhancement for gold nanodimer with different gap distance; *d* is the gap distance between the plasmonic individuals; (**b**) The per-sphere-weighted optical heating power for gold nanodimer with different gap distance; (**c**) Corresponding quantitative temperature fields for gold nanodimer with different gap distance at *t* = 5 ns; the laser irradiance is 0.15 mW/μm^2^; (**d**) Temperature distribution along the dotted lines in (**c**). The inset indicates the nonlinear temperature dependence on the interparticle distance.

One of the key factors that influence the EM and thermal coupling among the GNCs is the gap distance between the plasmonic individuals [37]. As indicated by Equations (2)–(4), the EM coupling is strongly sensitive to the relative position of the plasmonic individuals, thus resulting in the intensive dependence for the optical absorption cross sections of the GNCs on the gap distance. As simulated in Figure 4a, by taking the Au dimer as the example for GNCs, with the gap distance *d* decreasing from 2 nm to 0.5 nm, the collective resonance of the electric dipoles and a strong LSPR-induced electric field enhancement are observed. We quantitatively simulated the optical heating power per sphere under a laser irradiance of 0.15 mW/μm^2^ for the Au dimer with the gap distance to be 0.5 nm, 1 nm, and 2 nm, respectively. The results in Figure 4b show that with the gap distance decreasing, the per-particle-weighted peak optical absorption of the dimer grows nearly 47%, with an obvious red shift of the absorption peak. The enhanced optical absorption will certainly induce the increment of the temperature filed of the Au dimer. As quantitatively simulated in Figure 4c, when under the same laser irradiance, with the gap distance decreasing, the temperature of the Au dimer is nonlinearly amplified. Figure 4d shows the spatial temperature distribution for the Au dimer in Figure 4c. The temperature enhancement is about 59%, which is higher than that of the optical absorption enhancement, mainly contributed by the thermal coupling enhancement by decreasing the gap distance. These results indicate that by decreasing the gap distance between plasmonic individuals, the PA/PT effects can be nonlinearly enhanced.

**Figure 5:**
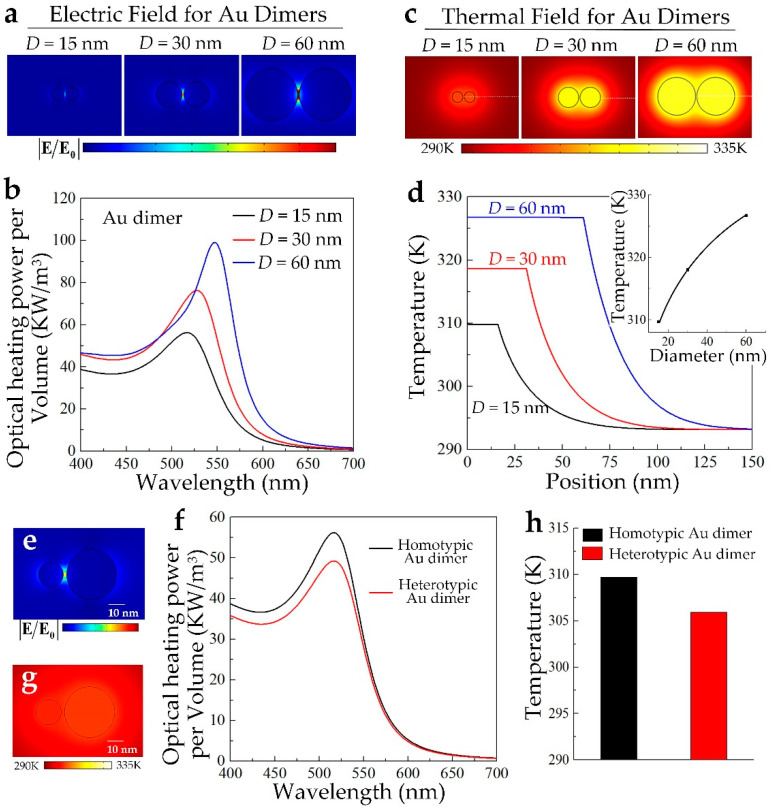
(**a**) LSPR-induced electric field enhancement for gold nanodimer with different particle sizes; *D* is the diameter of the plasmonic individuals; (**b**) Per-sphere-weighted optical heating power with a different particle size; the gap distance is 2 nm; (**c**) Corresponding quantitative temperature fields for the gold nanodimer with different particle size at *t* = 5 ns; the laser irradiance is 0.15 mW/μm^2^; (**d**) Temperature distribution along the dotted lines in (c). The inset indicates the nonlinear temperature dependence on the size of the individuals; (**e**) LSPR-induced electric field enhancement for heterotypic gold nanodimer; (**f**) The per-sphere-weighted optical heating power for heterotypic gold nano-dimer; (**g**) Corresponding quantitative temperature fields for the heterotypic gold nanodimer; the laser irradiance is 0.15 mW/μm^2^; (**h**) The quantitative temperature increment for homotypic and heterotypic gold nano-dimers.

The size of the individual is also a critical factor that strongly affects the nonlinear PA/PT effect of the GNCs. As shown in Figure 5a,b, by taking the Au dimer with the gap distance to be 2 nm as example, when irradiated by a laser pulse with an irradiance of 0.15 mW/μm^2^, as the diameter of the individual D increases, the simulated EM field is slightly enhanced, where the hot-spot phenomenon for the larger Au dimer becomes obvious. The quantitatively calculated peak optical heating power per volume of the Au dimer increases with the size. By further quantitatively simulating the temperature field for the Au dimer with a different individual size, we find that even under the same laser irradiance, the temperature field is nonlinearly enhanced with the size increasing, as shown in Figure 5c. Figure 5d is the spatial temperature distribution for the Au dimer in Figure 5c for a different individual size. These results demonstrate that by increasing the individual size, the nonlinear PA/PT effect can be enhanced. While in the practical applications, this size effect should be balanced because it also influences the biocompatibility [38]. The above discussion is established on the premise that the size of the individual in the GNCs is the same, where we can define this case as dimensionally homotypic GNCs. In Figure 5e–h, we provide a demo to quantitatively compare the nonlinear enhancement of the PA/PT effect for dimensionally homotypic and heterotypic GNCs. The heterotypic Au dimer is composed of two Au spheres with a diameter of 30 and 60 nm. The quantitatively simulated EM field distribution and the optical heating power per volume is presented in Figure 5e,f. The result in Figure 5f indicates that compared with the homotypic Au dimer with a diameter of 30 nm, the optical absorption capability of the heterotypic Au dimer becomes weak, which results in the corresponding temperature field decreasing, as shown in Figure 5g,h.

**Figure 6:**
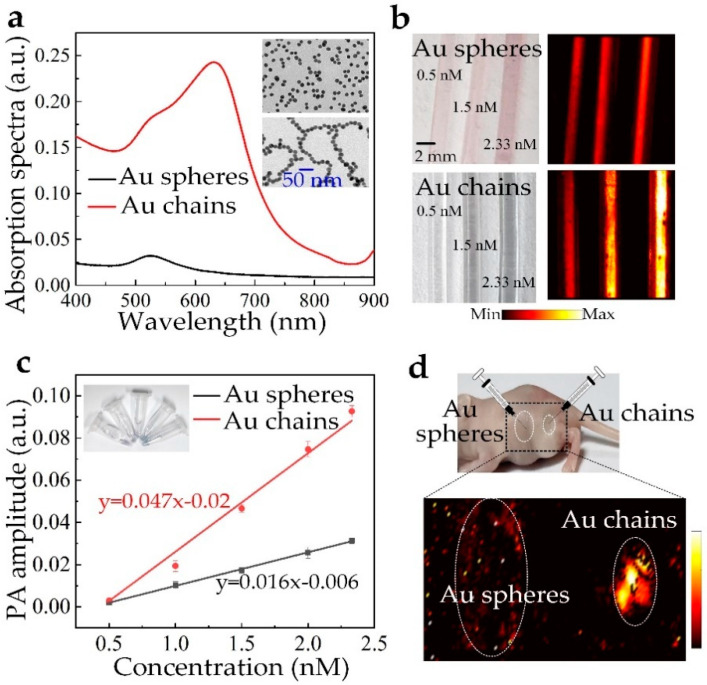
PA performance of GNCs and individual gold nanospheres. (**a**) UV absorbance spectra of gold nanospheres and GNCs at the same concentration; the inset is the transmission electron microscope (TEM) images of individual gold nanospheres (up) and GNCs (down); (**b**) PA imaging of the phantom gold nanospheres and GNCs; the concentrations for the samples are 0.5 nM, 1.5 nM, and 2.33 nM, respectively; (**c**) The linear dependence of the PA signal amplitude as a function of concentration for the GNCs; the inset is the photos of GNCs with different concentrations; (**d**) In vivo PA imaging of the gold nanospheres and nanochains on a mouse model.

To further demonstrate the nonlinear enhancement of the PA/PT effect for nanoassemblies, the PA performance of GNCs and the individual gold nanospheres are experimentally compared. The samples are brought from XFNANO Tech. Co., Ltd., Nanjing, China, and their concentrations are 2.4 nM. The two samples were firstly both diluted to 2.4 nM. Then, the two diluted samples were characterized by a transmission electron microscope (TEM) to show their morphologies. As shown in the inset of Figure 6a, TEM images shows that the gold nanospheres are uniformly distributed, and the GNCs are long chains, which are quite suitable to be used to validate our purpose. The size of the gold nanospheres and the individuals in the GNCs is about 17 nm. The per-particle-weighted optical absorption spectra for the two samples are shown in Figure 6a. It shows that the mass-weighted optical absorption of the GNCs is much larger than that of the gold nanospheres, with an obvious red shift of the absorption peak, which quite coincides with the simulations. Then, in order to experimentally demonstrate the nonlinear enhancement of the PA/PT effect for nanoassemblies, the two samples were prepared in three different concentrations (0.8, 1.2, 2.4 nM) to perform the PA experiments. As shown in Figure 6b, the PA image intensity for the two samples grows with the concentrations, where Figure 6c provides the linear dependence of the PA signal amplitude as a function of concentration for the GNCs. Importantly, even under the same mass concentrations, the GNCs show much higher image intensity compared with that of the gold nanospheres; this is mainly attributed to the nonlinear enhanced PA effect arising from the nanoassemblies. The experimental results coincide well with the simulations. Furthermore, in order to demonstrate the bioapplication of the nonlinearly enhanced PA effect by the nanoassemblies, gold nanospheres and nanochains are injected to in vivo mouse model, and then the PA experiment is immediately performed. The result in Figure 6d shows that the PA imaging intensity for the area of GNCs is much lighter than that of the gold nanospheres, indicating the great potential of the LSPR-mediated nanoassemblies for PA and PT applications.

## 3. Discussion

The self-assembly of nanoparticles has been of great interest to science and technology, both of which benefit from its ability to provide effective building blocks for physical, chemical, and biological applications. The rational design and construction of the nanoassemblies under theoretical guidance can effectively promote their applications in biological labeling, biochemical sensing, and biomedical imaging/therapy techniques [37]. Moreover, these nanoassemblies are also of tremendous potential to boost the applications in fields of accurate and reliable nanoscale manipulation and micromechanics, such as nanotweezers, microelectromechanical systems, and information storage devices [39].

This work demonstrates the nonlinearly enhanced PA/PT effect through constructing nanoassemblies by taking GNCs as example, where the strategy is also appliable for other metallic nanoparticles with other shapes [40]. Even though only a one-dimensional optical/thermal coupled nanochains is shown, this strategy can also be readily extended to two- or three-dimensional coupled nanoassemblies with much higher coupling efficiency, and a nonlinear PA/PT effect can be expected. With the number of the nanoparticles in the nanoassemblies increases, the coupling efficiency can become stronger; however, the size of the nanoassemblies should be considered in practical biological applications. Meanwhile, the structural stability of the nanoassemblies should be also considered in practical applications.

## 4. Conclusions

In conclusion, we quantitatively simulated the nonlinearly enhanced PA/PT effect by strong LSPR-coupled nanoassemblies. The strong optical and thermal coupling between the individual nanoparticles resulted in a several-time enhancement of the per-particle-weighted optical absorption, consequential thermal field enhancement, and initial PA pressure, compared with their individuals. Quantitative discussion about the dependence of the nonlinear PA/PT enhancement on the assembly chain length, the size of the individuals, the interparticle distance, and the size uniformity of the building blocks were provided. PA experiments on GNCs and individual gold nanospheres were performed to validate the proposition, and experimental results well silhouetted the theoretical discussion. Our work paves the way for the rational design and engineering of plasmonic nanoassemblies with improved PA/PT conversion efficiency.

## References

[1] Clavero C. (2014). Plasmon-induced hot-electron generation at nanoparticle/metal-oxide interfaces for photovoltaic and photocatalytic devices. Nat. Photonics.

[2] Ng S.M., Koneswaran M., Narayanaswamy R. (2016). A review on fluorescent inorganic nanoparticles for optical sensing applications. RSC Adv..

[3] Yang X., Yang M., Pang B., Vara M., Xia Y. (2015). Gold Nanomaterials at Work in Biomedicine. Chem. Rev..

[4] Jensen T.R., Malinsky M.D., Haynes C.L., Van Duyne R.P. (2000). Nanosphere lithography: Tunable localized surface plasmon resonance spectra of silver nanoparticles. J. Phys. Chem. B.

[5] Yu H., Peng Y., Yang Y., Li Z.-Y. (2019). Plasmon-enhanced light–matter interactions and applications. npj Comput. Mater..

[6] Hutter E., Fendler J.H. (2004). Exploitation of localized surface plasmon resonance. Adv. Mater..

[7] Xin H., Namgung B., Lee L.P. (2018). Nanoplasmonic optical antennas for life sciences and medicine. Nat. Rev. Mater..

[8] Brongersma M.L., Halas N.J., Nordlander P. (2015). Plasmon-induced hot carrier science and technology. Nat. Nanotechnol..

[9] Ding S.-Y., Yi J., Li J.-F., Ren B., Wu D.-Y., Panneerselvam R., Tian Z.-Q. (2016). Nanostructure-based plasmon-enhanced Raman spectroscopy for surface analysis of materials. Nat. Rev. Mater..

[10] Li W.W., Chen X.Y. (2015). Gold nanoparticles for photoacoustic imaging. Nanomedicine.

[11] Cheng X.J., Sun R., Yin L., Chai Z.F., Shi H.B., Gao M.Y. (2017). Light-Triggered Assembly of Gold Nanoparticles for Photothermal Therapy and Photoacoustic Imaging of Tumors In Vivo. Adv. Mater..

[12] Weber J., Beard P.C., Bohndiek S.E. (2016). Contrast agents for molecular photoacoustic imaging. Nat. Methods.

[13] Liu Y., Yin J.-J., Nie Z.H. (2014). Harnessing the collective properties of nanoparticle ensembles for cancer theranostics. Nano Res..

[14] Fan J.A., Wu C., Bao K., Bao J., Bardhan R., Halas N.J., Manoharan V.N., Nordlander P., Shvets G., Capasso F. (2010). Self-assembled plasmonic nanoparticle clusters. Science.

[15] Xing R., Liu K., Jiao T., Zhang N., Ma K., Zhang R., Zou Q., Ma G., Yan X. (2016). An injectable self-assembling collagen–gold hybrid hydrogel for combinatorial antitumor photothermal/photodynamic therapy. Adv. Mater..

[16] He J., Wei Z., Wang L., Tomova Z., Babu T., Wang C., Han X., Fourkas J.T., Nie Z. (2013). Hydrodynamically Driven Self?Assembly of Giant Vesicles of Metal Nanoparticles for Remote? Controlled Release †. Angew. Chem..

[17] Li Y., Liu G., Ma J., Lin J., Lin H., Su G., Chen D., Ye S.-F., Chen X., Zhu X. (2017). Chemotherapeutic drug-photothermal agent co-self-assembling nanoparticles for near-infrared fluorescence and photoacoustic dual-modal imaging-guided chemo-photothermal synergistic therapy. J. Controlled Release.

[18] Gutierrezwing C., Santiago P., Ascencio J.A., Camacho A., Joseyacaman M. (2000). Self-assembling of gold nanoparticles in one, two, and three dimensions. Appl. Phys. A Mater. Sci. Process..

[19] Mallidi S., Larson T., Tam J., Joshi P.P., Karpiouk A., Sokolov K., Emelianov S.Y. (2009). Multiwavelength Photoacoustic Imaging and Plasmon Resonance Coupling of Gold Nanoparticles for Selective Detection of Cancer. Nano Lett..

[20] Huang P., Lin J., Li W., Rong P., Wang Z., Wang S., Wang X., Sun X., Aronova M., Niu G. (2013). Biodegradable Gold Nanovesicles with an Ultrastrong Plasmonic Coupling Effect for Photoacoustic Imaging and Photothermal Therapy. Angew. Chem. Int. Ed..

[21] Liu Y., He J., Yang K., Yi C., Liu Y., Nie L., Khashab N.M., Chen X., Nie Z. (2015). Folding Up of Gold Nanoparticle Strings into Plasmonic Vesicles for Enhanced Photoacoustic Imaging. Angew. Chem. Int. Ed..

[22] Nam S.Y., Ricles L.M., Suggs L.J., Emelianov S.Y. (2012). Nonlinear photoacoustic signal increase from endocytosis of gold nanoparticles. Opt. Lett..

[23] Liu X., González M.G., Niessner R., Haisch C. (2012). Strong size-dependent photoacoustic effect on gold nanoparticles: A sensitive tool for aggregation-based colorimetric protein detection. Anal. Methods.

[24] Guerrero-Martínez A., Gómez J.L.A., Auguié B., Cid M.-M., Liz-Marzán L.M. (2011). From individual to collective chirality in metal nanoparticles. Nano Today.

[25] Groeneveld R.H.M., Sprik R., Lagendijk A. (1995). Femtosecond spectroscopy of electron-electron and electron-phonon energy relaxation in Ag and Au. Phys. Rev. B.

[26] Giustino F. (2017). Electron-phonon interactions from first principles. Rev. Mod. Phys..

[27] Furlani E.P., Karampelas I.H., Xie Q. (2012). Analysis of pulsed laser plasmon-assisted photothermal heating and bubble generation at the nanoscale. Lab Chip.

[28] Shi Y., Qin H., Yang S., Xing D. (2016). Thermally confined shell coating amplifies the photoacoustic conversion efficiency of nanoprobes. Nano Res..

[29] Draine B.T., Flatau P.J. (1994). Discrete-dipole approximation for scattering calculations. J. Opt. Soc. Am. A.

[30] Gunnarsson L., Rindzevicius T., Prikulis J., Kasemo B., Käll M., Zou S., Schatz G.C. (2005). Confined Plasmons in Nanofabricated Single Silver Particle Pairs: Experimental Observations of Strong Interparticle Interactions. J. Phys. Chem. B.

[31] Rioux D., Vallières S., Besner S., Munoz P., Mazur E., Meunier M. (2013). An Analytic Model for the Dielectric Function of Au, Ag, and their Alloys. Adv. Opt. Mater..

[32] Auguié B., Barnes W.L. (2008). Collective Resonances in Gold Nanoparticle Arrays. Phys. Rev. Lett..

[33] Hatef A., Darvish B., Dagallier A., Davletshin Y.R., Johnston W., Kumaradas J.C., Rioux D., Meunier M. (2015). Analysis of Photoacoustic Response from Gold–Silver Alloy Nanoparticles Irradiated by Short Pulsed Laser in Water. J. Phys. Chem. C.

[34] Calasso I.G., Craig W., Diebold G.J. (2001). Photoacoustic Point Source. Phys. Rev. Lett..

[35] Pelivanov I., Kopylova D.S., Podymova N.B., Karabutov A.A. (2009). Optoacoustic method for determination of submicron metal coating properties: Theoretical consideration. J. Appl. Phys..

[36] Kuttruff H. (2012). Ultrasonics: Fundamentals and Applications.

[37] Ghosh S.K., Pal T. (2007). Interparticle coupling effect on the surface plasmon resonance of gold nanoparticles: From theory to applications. Chem. Rev..

[38] Ekkapongpisit M., Giovia A., Follo C., Caputo G., Isidoro C. (2012). Biocompatibility, endocytosis, and intracellular trafficking of mesoporous silica and polystyrene nanoparticles in ovarian cancer cells: Effects of size and surface charge groups. Int. J. Nanomed..

[39] Zhao H., Chang M., Liu X., Gabayno J.L., Chen H.T. (2014). Design and implementation of shape memory alloy-actuated nanotweezers for nanoassembly. J. Micromech. Microeng..

[40] Sotiriou G.A., Starsich F., Dasargyri A., Wurnig M.C., Krumeich F., Boss A., Leroux J.-C., Pratsinis S.E. (2014). Photothermal Killing of Cancer Cells by the Controlled Plasmonic Coupling of Silica-Coated Au/Fe_2_O_3_ Nanoaggregates. Adv. Funct. Mater..

